# ﻿Two new species of *Pholiota* (Agaricales, Strophariaceae) from the southwest of China

**DOI:** 10.3897/mycokeys.109.133879

**Published:** 2024-10-08

**Authors:** Jia-hui Huang, Chun-yu Lei, Ya-lun Shen, En-jing Tian

**Affiliations:** 1 Engineering Research Center of Edible and Medicinal Fungi, Ministry of Education, Jilin Agricultural University, Changchun, 130118, China Jilin Agricultural University Changchun China

**Keywords:** Chrysocystidia, morphology, phylogeny, Subgenus *Pholiota*, taxonomy

## Abstract

Two new mushroom species from Southwest China, *Pholiotacylindrospora* and *P.subterrestris*, are described in this study. *Pholiotacylindrospora* is characterized by its dry pileus with slightly recurved and triangular scales, cylindrical basidiospores, and two types of pleurocystidia—leptocystidia and chrysocystidia—as well as its growth on soil. *Pholiotasubterrestris* is identified by a brownish-orange pileus with numerous brown fibrillose scales; pale brown lamellae with even edges; a stipe covered with recurved fibrillose scales; elliptical spores with a distinct but small germ pore; and pleurocystidia containing typical amorphous refractive inclusions of chrysocystidia. Both species are described and illustrated, and a phylogenetic analysis of a multigene dataset (ITS + 28S) is presented. Morphological and phylogenetic analyses confirm that *P.cylindrospora* and *P.subterrestris* are distinct from the other Pholiota species, and both belong to the subgenus Pholiota. A key to the species of subgenus Pholiota from China is provided.

## ﻿Introduction

*Pholiota* (Fr.) P. Kumm. is distributed worldwide, especially in the northern temperate zone, and currently contains approximately 150–160 species (Smith and Hesler 1968; Jacobsson 1991; Holec 2001; Kirk et al. 2008; Noordeloos 2011; Holec et al. 2014; He et al. 2019; Liu et al. 2024). Species of this genus grow on wood, sawdust, humus, soil, or, more rarely, on sphagnum beds or charcoal (Smith and Hesler 1968; Jacobsson 1991). Most *Pholiota* species are saprotrophic, and a few are parasitic (Holec 2001). Some species of this genus are edible and medicinal, such as *P.adiposa* (Batsch) P. Kumm. and *P.aurivella* (Batsch) P. Kumm. (Smith and Hesler 1968; Wu et al. 2019). However, this genus also contains mildly toxic species (Smith and Hesler 1968; Wu et al. 2019).

*Pholiota* is an important and stable genus of the family Strophariaceae. Nevertheless, its previously proposed infrageneric classification systems vary owing to differing concepts of the genus, from as many as seven subgenera to three (Singer 1962; Smith and Hesler 1968; Singer 1975; Jacobsson 1991; Holec 2001; Noordeloos 2011). Tian and Matheny (2021) proposed that *Pholiota* sensu stricto comprises at least two major subgenera, Subgen. Pholiota and Subgen. Flammuloides, although several residual poorly placed lineages were also noted, depending on the dataset analyzed in the study. The subgenus Pholiota is stable in different infrageneric taxonomic systems of the genus *Pholiota*. The subgenus Pholiota is mainly characterized by pileus and stipe with distinct scales, as well as chrysocystidia or very similar sterile cells in the hymenium of many of the species (Smith and Hesler 1968; Holec 2001). Approximately 62 species of *Pholiota* have been reported in China, including 12 species belonging to the subgenus Pholiota (Mao and Jiang 1993; Tian and Bau 2004; Bau et al. 2005; Tian and Bau 2011a, 2011b; Tian et al. 2016; Liu et al. 2024).

This study discovered two new species from the Southwest of China, belonging to subgenus Pholiota, based on morphological examination and phylogenetic analyses. The results are presented in the following sections.

## ﻿Materials and methods

### ﻿Morphological studies

Fresh specimens were collected from Southwest China and deposited at the Herbarium of Mycology of Jilin Agricultural University (HMJAU) in Changchun City, China. The specimens were documented using color descriptions from Kornerup and Wanscher (1978) and then dried on dehydrators. The dried specimens were examined in 5% KOH solution, and the dextrinoid reactions were tested using Melzer’s reagent (Singer 1986).

Basidiospores were measured and examined according to the methods of Tian and Matheny (2021) and Tian et al. (2021). Line drawings of the microstructures were obtained from the rehydrated samples.

Abbreviations: “L” denotes the number of lamellae extending to the stipe; “I” indicates the number of lamellae between each adjacent pair; “Q” represents the length/breadth ratio of basidiospores; “Q_m_” refers to the mean value of “Q” measurements across all studied collections ± the sample standard deviation.

### ﻿DNA extraction, PCR, and data set assembly

DNA was extracted from 10 to 20 mg of ground, dried basidiome tissue using a Plant Genomic DNA Kit (Tiangen Biotech Co., Ltd, Beijing). The internal transcribed spacer region (ITS) of Nuc rDNA was amplified and sequenced using the primers ITS1 (TCCGTAGGTGAACCTGCGG) and ITS4 (TCCTCCGCTTATTGATATGC) (White et al. 1990; Gardes and Bruns 1993). The 28S rDNA ribosomal region was amplified and sequenced using the primers LR0R (GTACCCGCTGAACTTAAGC) and LR7 (TACTACCACCAAGATCT) (Vilgalys and Hester 1990). The DNA sequences generated in this study were deposited in GenBank. A two-locus dataset (ITS+28S) was assembled using AliView 1.28 (Larsson 2014). The sequences generated in this study were merged with the partial multigene dataset from Tian and Matheny (2021). The ITS+28S dataset was constructed using representative *Pholiota* species, including 36 type collections. *Stropharia* species were selected as outgroups, based on Tian and Matheny (2021). Information on the sequences used in this study is shown in Table 1.

**Table 1.** Specimen data and DNA sequences analyzed in this study.

Species	Specimen-voucher (Herbarium)	Origin	GenBank accession numbers	
			ITS	28S
*Pholiotaadiposa*	ET37 (HMJAU37520)	China, Jilin	MN209721	MN251112
*P.agglutinata*	AHS60691 (TENN-F-028806, isotype)	USA, Idaho	MN149356	MN251113
*P.aurivella*	CBS 262.32	Netherlands	MH855317	–
*P.aurivella*	SJ84131	Sweden	AF195603	–
*P.aurivella*	ET27 (HMJAU37516)	China, Jilin	MN209728	MN251116
*P.aurivella*	ET42 (HMJAU37521)	China, Jilin	MN209729	MN251117
*P.aurivella*	ET19 (HMJAU37425)	China, Inner Mongolia	MN209771	MN251154
*P.avellaneifolia*	AHS59589 (TENN-F-028809 isotype)	USA, Idaho	MN209731	MN251120
*P.baeosperma*	TFB7383 (TENN-F-054431)	Chile	MG735312	–
*P.baeosperma*	RHP8315 (TENN-F-054993)	Argentina	KY559332	–
*P.baptistii*	ET542 TENN-F-028810 (isotype)	USA, Idaho	MN149364	–
*P.brunnescens*	AHS3525 (MICH 11657, holotype)	USA, Oregon	MG735292	–
*P.brunnescens*	PBM3057 (TENN-F-063855)	USA, California	MG735314	–
*P.caespitosa*	TENN-F-015908 (holotype)	USA, Tennessee	NR119908	–
*P.carbonaria*	AHS9500 (MICH 11663, holotype)	USA, California	MG735288	–
*P.castanea*	DPL7769 (TENN 071878)	USA, Texas	MH016952	–
*P.castanea*	TENN 020269 (holotype)	USA, Tennessee	HQ222025	–
*P.chocenensis*	PRM 895066 (holotype)	Czech Republic	NR_155622	–
*P.conissans*	SJ96017, FCUG1273	Sweden	AF195606	–
*P.conissans*	CBS 175.47	France	MH856205	–
*P.conissans*	CBS 243.50	France	MH856603	–
*P.conissans*	voucher 395	Italy	JF908575	–
*P.conissans*	voucher 6610	Italy	JF908581	–
***P.cylindrospora***	**ET-Ti2(holotype)**	**China, Yunnan**	**PQ013666**	**PQ013732**
***P.cylindrospora***	**ET-yun2**	**China, Yunnan**	**PQ013686**	**PQ013733**
*P.decorata*	AHS54770 (TENN-F-028816)	USA, Idaho	MN209734	–
*P.ferrugineolutescens*	TENN-F-028807 (isotype)	USA, California	HQ222026	–
*P.flavescens*	TENN-F-01309) (isotype)	USA, Tennessee	MN209735	MN251124
*P.fulviconica*	LRH28818 (TENN-F-028818)	USA, Idaho	MN209738	MN251126
*P.fulviconica*	AHS65898 (TENN-F-028820, **i**sotype)	USA, Idaho	MN209739	MN251127
*P.fulvozonata*	AHS73887 (MICH 5316, holotype)	USA, Idaho	MG735290	–
*P.gallica*	PRM 933232	France	LN889967	–
*P.gallica*	MPU 3478 (holotype)	France	HG007988	–
*P.gummos*	SJ84095, FCUG1254	Sweden	AF195605	–
*P.gummos*	CBS 216.39	Portugal	MH855985	–
*P.gummos*	CBS 210.48	Portugal	MH856313	–
*P.highlandensis*	NYS 1468.1 (isotype)	USA, New York	MH016956	–
*P.highlandensis*	PBM4085 (TENN-F-071544)	USA, Tennessee	MG735310	–
*P.humii*	AHS58633 (TENN-F-028822, isotype)	USA, Idaho	MN209740	MN251128
*P.jahnii*	SJ83118, FCUG1061	Sweden	AF195604	–
*P.kodiakensis*	TENN-F-028804 (isotype)	USA, Alaska	MN149360	–
*P.lenta*	ET33 (HMJAU37519)	China, Jilin	MN209742	MN251130
*P.lenta*	PBM4233 (TENN-F-074640)	USA, N. Carolina	MN209743	MN251131
*P.limonella*	ET28 (HMJAU37345)	China, Inner Mongolia	MN209747	MN251135
*P.limonella*	MF68025 (TENN-F-074669)	USA, Tennessee	MN209748	MN251136
*P.limonella*	ET11 (HMJAU37362)	China, Jilin	MN209741	MN251129
*P.limonella*	ET13 (HMJAU37514)	China, Jilin	MN209746	MN251134
*P.lubrica*	ET29 (HMJAU37517)	China, Jilin	MN209751	MN251139
*P.lubrica*	ET4 (HMJAU22678)	China, Jilin	MN209753	MN251141
*P.lundbergii*	LL950724	Sweden	AF195607	–
*P.lurida*	AHS66386 (TENN-F- 028770, isotype)	USA, Michigan	MN209757	–
*P.luteobadia*	AHS43222 (MICH 11688, holotype)	USA, Michigan	MG735289	–
*P.marangania*	HLepp856 (CANB 574576)	Australia	MG735320	–
*P.melliodora*	AHS68780 (TENN-F-028861, paratype)	USA, Oregon	MN209758	–
*P.mixta*	SJ96022	Sweden	AF195609	–
*P.mixta*	PBM2499 (TENN-F-062357)	USA, Mass.	MH016953	–
*P.molesta*	AHS65008 (TENN-F-028830, isotype)	USA, Idaho	MG735296	–
*P.molesta*	JFA9246 (WTU 10719)	USA, Washington	MG735297	–
*P.multicingulata*	PBM3587 (TENN-F-066655)	Australia	MN209760	–
*P.multicingulata*	ET23 (HMJAU37414)	China, Yunnan	MN209761	MN251146
*P.nameko*	ET7 (HMJAU37512 / TENN-F-074767)	China, Jilin	MN209762	MN251147
*P.nameko*	ET10 (HMJAU22620 as *Pholiota* “*microspora*”)	China, Jilin	MN209759	MN251145
*P.occidentalis*	AHS58470 (TENN-F-028874, paratype)	USA, Idaho	MN209765	MN251150
*P.olivaceodisca*	TENN-F-017778 (holotype)	USA, Tennessee	NR119909	–
*P.olivaceophylla*	MICH 290502 (holotype)	USA, CA	KF878381	–
*P.parvula*	AHS42234 (TENN-F-028834, isotype)	USA, Michigan	MN149362	–
*P.polychroa*	PBM2866 (TENN-F-062649)	USA, Louisiana	MG735317	–
*P.prolixa*	AHS5027 (TENN-F-028838, isotype)	USA, Michigan	MN209766	–
*P.rubronigra*	AHS56192 (TENN-F-028840, isotype)	USA, California	MH016955	–
*P.rufodisca*	B925 (TENN-F-028869, paratype)	USA, New Mexico	MN209767	–
*P.* sp.	ET38 (HMJAU22691)	China, Jilin	MN209737	MN251125
*P.spumosa*	ET12 (HMJAU37513)	China, Jilin	MN209776	MN251159
*P.spumosa*	LRH12950 (TENN-F-012950	USA, Tennessee	MN149361	–
*P.squarrosa*	PBM2735 (TENN-F-062547)	USA, Colorado	DQ494683	DQ470818
*P.squarrosa*	AH-48188	Spain	MF345959	–
*P.squarrosa*	SFC20140912-I01	South Korea	KX773886	–
*P.squarrosa*	Voucher 28	Germany	FR686575	–
*P.squarrosa*	ET26 (HMJAU37515)	China, Jilin	MN209777	MN251160
*P.squarrosa*	ET15 (HMJAU37366)	China, Jilin	MN209778	MN251161
*P.squarrosoides*	TENN-F-061728	USA, Tennessee	FJ596877	–
*P.squarrosoides*	SFC20120814-45	South Korea	KX773887	–
*P.squarrosoides*	JACA-MICO-00132	Spain	MF345957	–
*P.squarrosoides*	TENN-F-061692	USA, N. Carolina	FJ596860	–
*P.squarrosoides*	AH-48186	France	MF345958	–
*P.stratosa*	AHS64684 (TENN-F-028845, isotype)	USA, Michigan	MN209779	–
*P.subsaponacea*	AHS74095 (MICH 5332, holotype)	USA, Idaho	MG735287	–
***P.subterrestris***	**ET-072240 (holotype)**	**China, Guizhou**	**PQ013700**	**PQ013730**
***P.subterrestris***	**ET-gui2**	**China, Guizhou**	**PQ013701**	**PQ013731**
*P.tennesseensis*	TENN-F-018848 (holotype)	USA, Tennessee	NR_119910	–
*P.terrestris*	RAS371 (TENN-F-074807)	USA, Tennessee	MN209781	–
*P.terrestris*	iNat62825743	USA,New York	OP681747	–
*P.terrestris*	iNat62849835	USA,New York	MW644574	–
*P.tetonensis*	WGS3763 (TENN-F-028849, isotype)	USA, Wyoming	MN149367	–
*P.velaglutinosa*	AHS9285 (TENN-F-028851, isotype)	USA, Oregon	MH016954	–
*P.virescens*	HMJAU22498 (holotype)	China, Jilin	JF961378	–
*P.virescentifolia*	TENN-F-020591 (holotype)	USA, Tennessee	NR_119911	–
*P.virgata*	B763 (TENN-F-028832, paratype)	USA, New Mexico	MN209782	–
*Strophariaacanthostipitata*	CA01222011 (TENN-F-071898)	Dominican Republic	MG735313	–
*S.acanthostipitata*	JBSD 127401(holotype)	Dominican Republic	NR_156637	MF882994
*S.rugosoannulata*	X-31	USA	KC176328	–

Note: DNA sequences generated in this study are in bold.

### ﻿Phylogenetic analysis

Bayesian Inference (BI) analysis was conducted using MrBayes 3.2.7 (Ronquist et al. 2012). Maximum Likelihood (ML) and bootstrap analyses were performed in RAxML 8.2.9 (Stamatakis 2014). GTR+I+G was selected as the best-fit model for the datasets using jModelTest 2 (Guindon and Gascuel 2003; Darriba et al. 2012). One thousand bootstrap replicates were performed in ML analysis for the dataset. Two million generations were run in the BI analysis using four chains and other default parameters for the dataset, sampling trees, and other parameters every 1000 generations. Trees sampled from the first 25% of generations were discarded as burn-in, by which point the average standard deviation of the split frequencies had reached < 0.01. After the BI analysis, the potential scale reduction factors (PSRFs) were approximately 1.0 for all parameters. The Bayesian Posterior Probabilities (BPPs) were then calculated. Alignments of the datasets were submitted to TreeBASE (31577).

## ﻿Results

### ﻿Phylogeny

**Figure 1.**
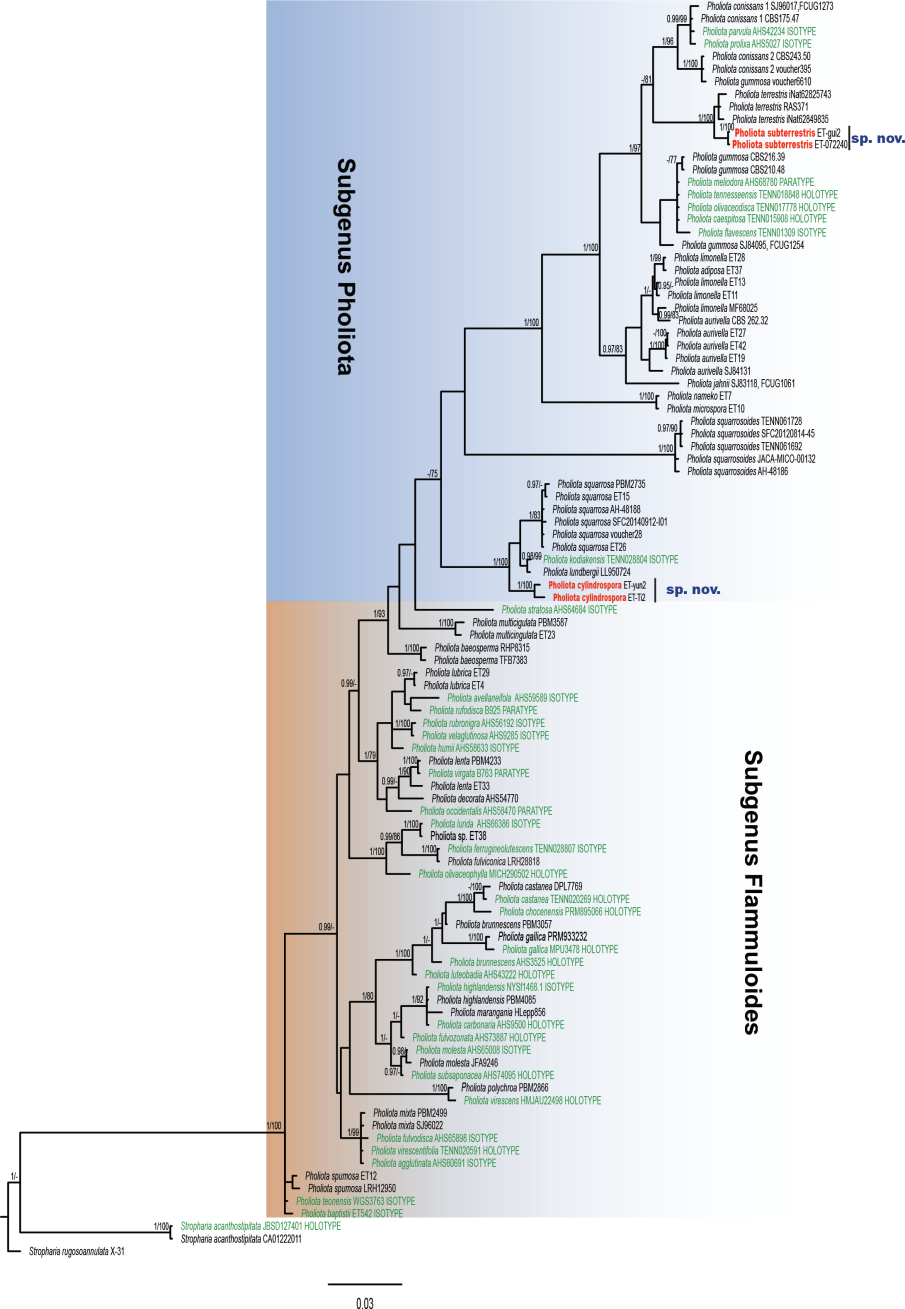
BI phylogram of *Pholiota* based on the dataset (ITS+28S). PPs > 0.95 and bootstrap values > 70% are shown. Types are indicated in green, and the new species from China in this study are in red.

Eight new sequences (four ITS and four 28S) from the two species were produced in this study (Table 1). The dataset (ITS+28S) consisted of 59 taxa (36 type collections) and 2922 sites, all of which were included before analyses. The phylogram, with branch lengths inferred from MrBayes including support values (PPs and bootstraps) from both BI and ML, is shown in Fig. 1. The ML analysis produced nearly identical estimates of the topology to those of BI.

In the phylogram (Fig. 1), the representative *Pholiota* species and type collections were gathered together with strong support (PP = 1, BP = 100), and two major clades were inferred: the Subgenus Pholiota clade and the Subgenus Flammuloides clade. The two new species described here, *P.cylindrospora* and *P.subterrestris*, clustered within the SubgenusPholiota and represented relatively independent lineages.

### ﻿Taxonomy

#### ﻿ Pholiota cylindrospora

Taxon classificationFungiAgaricalesStrophariaceae

E.J. Tian sp. nov.

2FA4D09F-8A9E-589F-BBA7-CAE151FF4D93

854827

Figs 2 , 3

##### Diagnosis.

Differs from other *Pholiota* species by dry pileus with slightly recurved and triangular scales, cylindrical basidiospores, and two types of pleurocystidia, including leptocystidia and chrysocystidia, as well as growing on soil.

**Figure 2.**
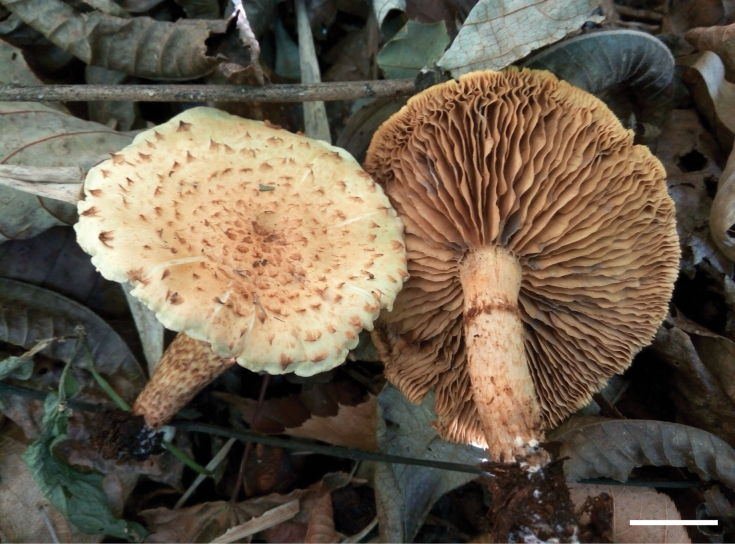
Basidiomata of *Pholiotacylindrospora* (HMJAU37432, holotype). Photo by Shi-liang Liu. Scale bar: 1 cm.

##### Holotype.

China. • Yunnan, Baoshan City, Gaoligong Mountains, Baihualing; elev. 910 m; 25°03'33′′N, 98°49'18′′E; scattered on soil in broad-leaved forest; 30 November 2015; Shi-liang Liu 37432 (holotype: HMJAU!).

**Figure 3.**
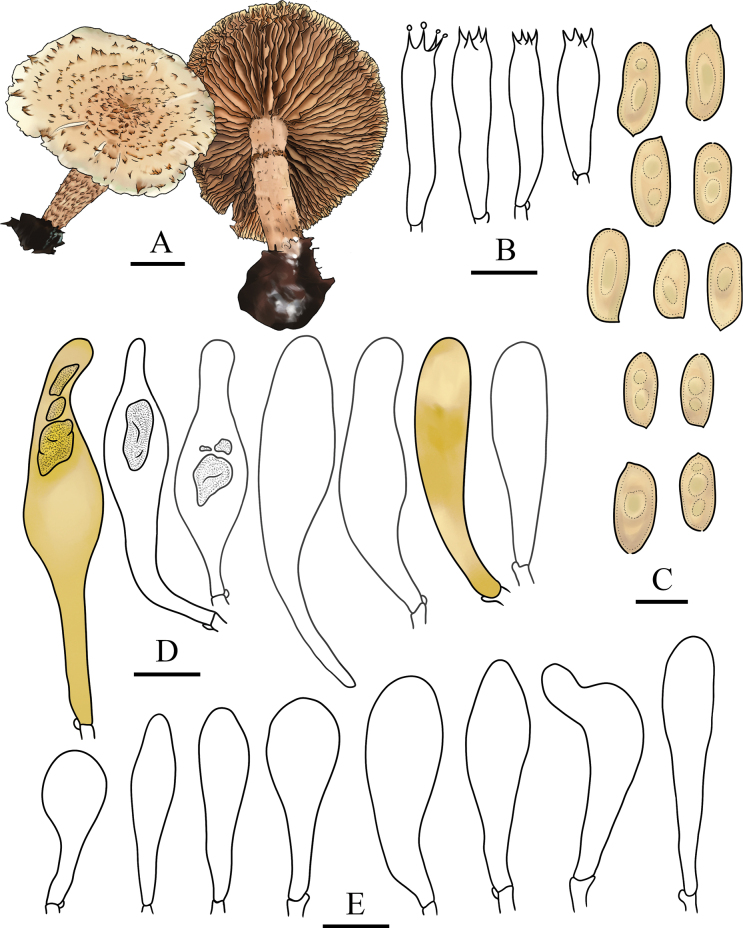
Microcharacters of *Pholiotacylindrospora* (HMJAU37432, holotype). Drawings by Jia-hui Huang **A** basidiocarps **B** basidia **C** basidiospores **D** pleurocystidia **E** cheilocystidia. Scale bars: 1 cm (**A**); 5 µm (**C**); 10 µm (**B**, **D**, **E**).

##### Etymology.

Referring to the cylindric basidiospores.

##### Description.

***Pileus*** 50–70 mm in diameter, convex to broadly convex, becoming nearly plane, with a low obtuse umbo; surface dry, pale orange to apricot (5A3–5B6), paler towards the margin, decorated with concentric, slightly recurved, triangular, light brown (6D7) scales. ***Context*** pale (2A2), odor and taste mild. ***Lamellae*** adnate, broad, moderately close, L = 38–42, I = 2–6, pallid at first, becoming brown (6D6), the edges waved. ***Stipe*** 45–60 mm long, 8–11 mm thick, central, equal to tapered towards the base, solid, ground color pallid, smooth to silky above an annular zone, towards the base with yellowish brown to brown (5D6–6D6) multizonate squamules or sometimes with scattered brown (6D5) fibrils, with white mycelium at the base.

***Basidiospores*** (6.5–)7.0–9.5(–10.0) × 3.0–4.0 µm, Q = 1.75–2.83, Q_m_ = 2.30, in face view oblong to cylindric, sometimes crooked, in profile cylindric to slightly inequilateral, wall smooth and thin, germ pore very minute to not evident, pale rusty to yellowish brown (6E8–5E8) in KOH, slightly paler in Melzer’s reagent. ***Basidia*** 19–27 × 4.9–6.5 µm, 4-spored, clavate, hyaline in KOH. ***Pleurocystidia*** of two types: 1) leptocystidia, 35–55 × 8.5–10 µm, clavate to subfusoid, thin-walled, smooth, content homogeneous, hyaline to pale yellowish brown to brownish (5D5–5E7) in KOH; 2) chrysocystidia, 39–60 × 9.5–12.5 µm, lageniform to clavate with a rostrate to mucronate apex, rarely forked near the apex, with an amorphous highly refractive inclusion, pale rusty to yellowish brown (6E8–5D5) in KOH. ***Cheilocystidia*** 23–39 × 6–11 µm, fusiform, clavate to subcapitate at apex, wall thin and smooth, content homogeneous, hyaline, pale yellow to yellow brownish (4A3–5C7) in KOH. ***Caulocystidia*** not observed. ***Gill trama*** of parallel hyaline to yellowish white (4A2) hyphae in KOH and with smooth walls, the cells inflated, up to 23 µm in diam. ***Pileipellis*** a cutis of brownish yellow to light brown (5C8–5D5) hyphae 4.5–11 µm in diam., thin-walled, slightly incrusted to asperulate. ***Clamp connections*** present in all the tissues.

##### Habitat.

Scattered on soil in broad-leaved forest in late autumn.

##### Additional materials examined.

China. • Yunnan: Tengchong City, Yunfeng Mountain, on soil; 15 November 2019; Xiao-ming Zhang 37433 (HMJAU).

##### Discussion.

Cylindrical basidiospores are uncommon in the genus *Pholiota*, making this species easily distinguishable from the others. In addition, it is readily recognized because of its dry pileus with slightly recurved and triangular scales and two types of pleurocystidia, including leptocystidia and chrysocystidia, as well as its growth on soil.

The stipe with multizonate squamules of this species reminds one of *Pholiotamulticingulata* Horak, but the latter has smaller basidiospores (6.5–8 × 4.5–5 µm) and lacks chrysocystidia (Horak 1983).

*Pholiotacylindrospora* is similar to *P.squarrosa* (Vahl) P. Kumm. Both species have a dry pileus with obvious scales and two types of pleurocystidia: leptocystidia and chrysocystidia. However, the latter has smaller (6–7.5 × 3.8–4.5 µm) and elliptic basidiospores with a distinct germ pore and is wood-inhabiting (Smith and Hesler 1968; Holec 2001). *P.kodiakensis* A.H. Sm. & Hesler with two types pleurocystidia is also similar to *P.cylindrospora*; however, the former can be distinguished from the latter by its shorter spores (5–6 × 3–3.5 µm) and distinct germ pores with truncate apex (Smith and Hesler 1968). Furthermore, in the phylogram, *P.cylindrospora* clustered in the SubgenusPholiota clade (Fig. 1). In this clade, this species was sister to *P.kodiakensis* and *P.squarrosa* with high statistical support (PP = 1, BS = 100), but represented a relatively independent lineage (Fig. 1). Therefore, it is proposed here as a new species belonging to PholiotasubgenusPholiota based on morphological examination and phylogenetic analyses.

#### ﻿ Pholiota subterrestris

Taxon classificationFungiAgaricalesStrophariaceae

E.J. Tian & J.H. Huang sp. nov.

A851963F-837C-5D96-AF59-CE8AB27BCF96

854828

Figs 4 , 5

##### Diagnosis.

*Pholiotasubterrestris* is distinguished from the other species of the genus *Pholiota* by brownish orange pileus with numerous brown fibrillose scales, pale brown and moderately broad lamellae with even edges, stipe covered with recurved fibrillose scales, evanescent fibrillose annulus; elliptic spores with distinct but small germ pore, fusoid ventricose pleurocystidia with typical amorphous refractive inclusion of chrysocystidia.

**Figure 4.**
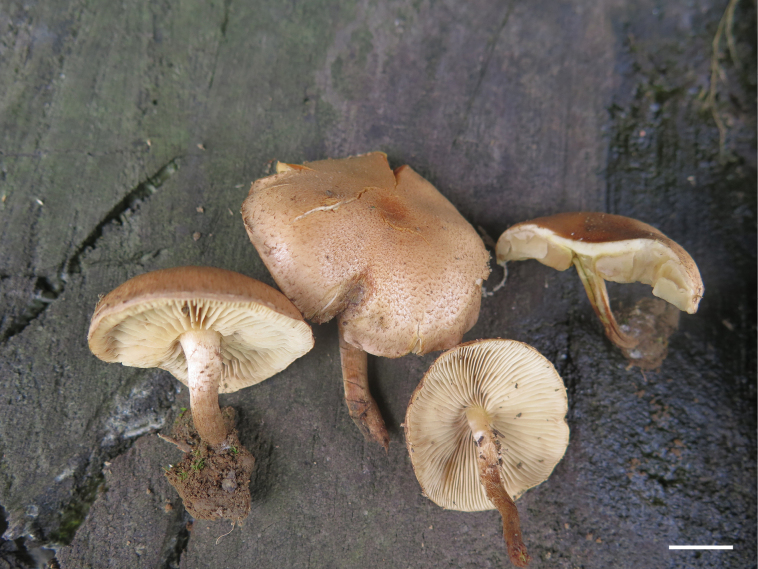
Basidiomata of *Pholiotasubterrestris* (HMJAU37434, holotype). Photo by En-jing Tian. Scale bar: 1 cm.

##### Holotype.

China. • Guizhou: Bijie City, Nayong County, Dapingqing National Wetland Park; elev. 1990 m; 26°41'10′′N, 105°27'30′′E; scattered on soil at base of stump; 22 July 2020; En-jing Tian 37434 (holotype: HMJAU!).

**Figure 5.**
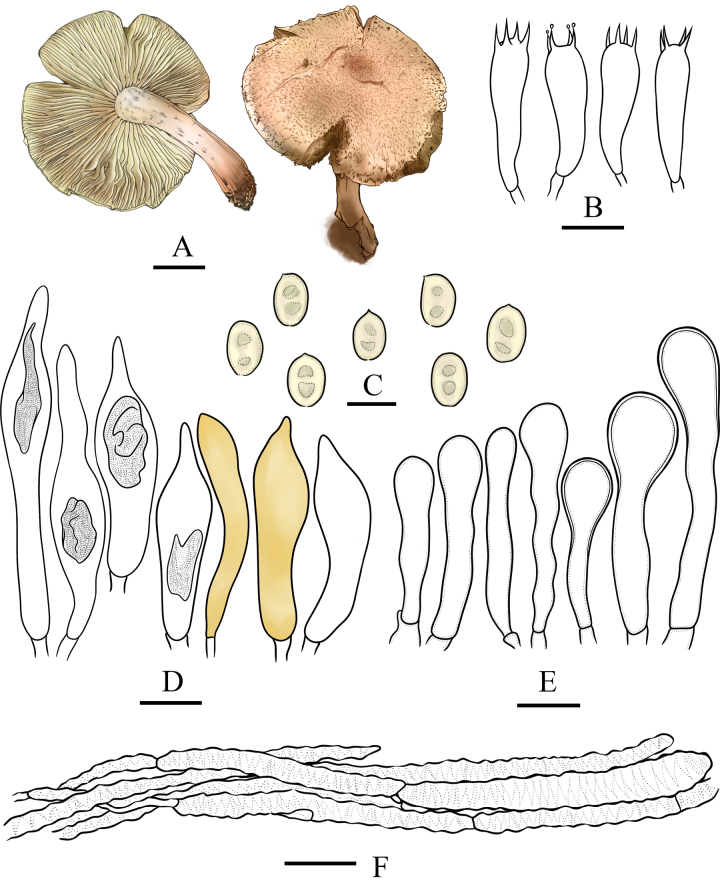
Microcharacters of *Pholiotasubterrestris* (HMJAU37434, holotype). Drawings by Jia-hui Huang **A** basidiocarps **B** basidia **C** basidiospores **D** pleurocystidia **E** cheilocystidia **F** pileipellis. Scale bars: 1 cm (**A**); 5 µm (**C**); 10 µm (**B**, **D**, **E**); 20 µm (**F**).

##### Etymology.

The epithet *subterrestris* refers to the similarity to *Pholiotaterrestris*.

##### Description.

***Pileus*** 10–35 mm in diameter, subhemispherical when young, with involute margin, slightly spread when mature, surface slightly viscid when wet, greyish orange to brownish orange (5B4–6C6), center dark, becoming paler towards the margin, covered with brown (6E7) fibrillose scales. ***Context*** thick, white to yellowish white (1A1–2A2), mild odor and taste. ***Lamellae*** decurrent to adnate or adnexed, moderately broad, close, L = 39–44, I = 3–7, pale yellow to light brown (4A3–5D6), edges even. ***Stipe*** 18–30 mm long, 4–7 mm thick, central, equal, solid, brownish orange (5C4) above, brown (6E5) below, covered with light brown (6D6) fibrillose squamulose, often compressed at the base

***Basidiospores*** 5.4–6.1 × 3.6–4.2 µm, Q = 1.30–1.56, Q_m_ = 1.47, smooth, in face view broadly elliptic to elliptic, in profile a little bean-shaped, apical pore distinct but small, containing oil-bearing droplet or irregular inclusions, greyish yellow (4B5) in KOH and light yellow (4A5) in Melzer’s reagent. ***Basidia*** 17.1–21.8 (–24.0) × 5.0–6.3 µm, 4-spored, clavate, hyaline in KOH, pale yellow (4A3) in Melzer’s reagent. ***Pleurocystidia*** of two types: 1) 33.3–46.7 × 5.5–9.5 µm, subclavate to fusoid ventricose with a subacute apex, wall thin and smooth, content homogeneous, blond to dark yellow (4C4–4C8) to hyaline in KOH; 2) chrysocystidia, 34.2–58 × 8.3–10.4 µm, fusoid to fusoid-ventricose, the neck often drawn out to a crooked filamentose projection with a subacute apex (up to 19.6 µm long), walls thin, smooth, with a refractive-amorphous inclusion, hyaline in KOH. ***Cheilocystidia*** abundant, (20.0–) 22.9–39.2 (–48.3) × 3.9–12.7 µm, clavate to clavate-capitate, wall thin, at times somewhat thickened in apex (about 1 µm), hyaline in KOH. ***Caulocystidia*** none observed. ***Gill trama*** of parallel hyphae, the cells inflated up to 27.5 µm, thin-walled, smooth, hyaline in KOH. ***Pileipellis*** hyphae hyaline to light brown (6D7), 3.7–10 µm diam., with encrusted walls. ***Content hyphae*** hyaline, 10–37.5 (–50) µm diam., cells inflated, smooth and thin-walled. ***Clamp connections*** present in all the tissues.

##### Habitat.

Scattered or gregarious on soil at the base of a stump or on buried wood in summer.

##### Additional materials examined.

China. • Guizhou: Bijie City, Qixingguan District, Baima Mountain, gregarious on soil or on buried wood, 15 August 2022, Guang-cheng Cao 37435 (HMJAU).

##### Discussion.

This species is characterized by a brownish orange pileus with numerous brown fibrillose scales, pale brown lamellae with even edges, a stipe covered with recurved fibrillose scales, an evanescent fibrillose annulus, elliptic spores with a distinct but small germ pore, and fusoid ventricose pleurocystidia with typical amorphous refractive inclusions of chrysocystidia.

*Pholiotasubterrestris* is similar to *P.terrestris* Overh., especially in terms of micro-characteristics, such as pleurocystidia, cheilocystidia, and basidiospores. However, it is easy to differentiate between the two species based on the macro characteristics. *P.terrestris* has a larger pileus (20–80 mm broad) without an orange tone, narrow and crowded lamellae with uneven edges, and a longer stipe (30–80 mm long) (Smith and Hesler 1968). Furthermore, in habitat, *P.terrestris* is caespitose on soil, whereas *P.subterrestris* is scattered or gregarious. Phylogenetic analyses showed that *P.subterrestris* was sister to *P.terrestris* in the SubgenusPholiota clade (Fig. 1), which also indicated a close relationship between the two species.

### ﻿Key to species of PholiotaSubgenusPholiota from China

**Table:** 

1	Pileus dry	**2**
–	Pileus viscid	**4**
2	Spores cylindric, germ pore very minute to not evident	***Pholiotacylindrospora* sp.nov. **
–	Spores elliptic, germ pore distinct	**3**
3	Spores 6–7.5 × 3.8–4.5 µm, germ pore apex not truncate	***P.squarrosa***
–	Spores 5–6 × 3–3.5 µm, germ pore apex truncate	***P.kodiakensis***
4	Pileus ground color white to whitish	***P.squarrosoides***
–	Pileus ground color not white to whitish	**5**
5	Spores 5–8 µm wide	**6**
–	Spores 2.5–5 µm wide	**7**
6	Spores 6–8 µm wide, germ pore apex truncate	***P.aurivelloides***
–	Spores 5–6 µm wide, germ pore apex not truncate	***P.aurivella***
7	Pileus with fibrillose scales	**8**
–	Pileus with spot-like or triangular scales	**10**
8	Pileus and stipe brilliant yellow, spores smaller (4–5 × 2.5–3 µm)	***P.flammans***
–	Pileus darker, spores bigger (> 4.5 µm long, > 3.5 µm wide)	**9**
9	Pileus 10–35 mm broad with orange tone, lamellae with even edges, stipe 18–30 mm long	***P.subterrestris* sp.nov.**
–	Pileus 20–80 mm broad without orange tone, lamellae with uneven edges, stipe 30–80 mm long	***P.terrestris***
10	Spores 5–6 µm long	***P.adiposa***
–	Spores 6–8 µm long	**11**
11	Lamellae whitish when young and becoming ferruginous	***P.limonella***
–	Lamellae yellow or pallid brownish when young	**12**
12	Lamellae pallid brownish when young and becoming cinnamon, germ pore distinct	***P.abietis***
–	Lamellae yellow when young, germ pore minute or inconspicuous	**13**
13	Stipe with appressed fibrillose scales, annulus persistent	***P.filamentosa***
–	Stipe with recured floccose scales, annulus evanescent	***P.squarrosoadiposa***

## Supplementary Material

XML Treatment for Pholiota cylindrospora

XML Treatment for Pholiota subterrestris

## ﻿Additional information

### Author ORCIDs

Jia-hui Huang https://orcid.org/0009-0001-0323-3446

Chun-yu Lei https://orcid.org/0009-0001-4275-6813

Ya-lun Shen https://orcid.org/0009-0000-7394-4457

En-jing Tian https://orcid.org/0000-0001-9742-7569

### Data availability

The research data used in this study can be found in GenBank (https://www.ncbi.nlm.nih.gov) and MycoBank (https://www.mycobank.org).
